# A Comparative Study of the Properties of Gelatin (Porcine and Bovine)-Based Edible Films Loaded with Spearmint Essential Oil

**DOI:** 10.3390/biomimetics8020172

**Published:** 2023-04-21

**Authors:** Saurabh Bhatia, Ahmed Al-Harrasi, Muhammad Jawad, Yasir Abbas Shah, Mohammed Said Al-Azri, Sana Ullah, Md Khalid Anwer, Mohammed F. Aldawsari, Esra Koca, Levent Yurdaer Aydemir

**Affiliations:** 1 Natural and Medical Sciences Research Center, University of Nizwa, Birkat Al Mauz, P.O. Box 33, Nizwa 616, Oman; 2 School of Health Science, University of Petroleum and Energy Studies, Dehradun 248007, India; 3 Center for Transdisciplinary Research, Department of Pharmacology, Saveetha Dental College and Hospital, Saveetha Institute of Medical and Technical Sciences, Saveetha University, Chennai 600077, India; 4 Department of Pharmaceutics, College of Pharmacy, Prince Sattam Bin Abdulaziz University, Al-Kharj 11942, Saudi Arabia; 5 Department of Food Engineering, Adana Alparslan Turkes Science and Technology University, 01250 Adana, Turkey

**Keywords:** gelatin, porcine, bovine, spearmint essential oil, edible films, active packaging

## Abstract

Gelatin (bovine/porcine)-based edible films are considered as an excellent carrier for essential oils (EOs) to preserve food quality and extend their shelf life. Spearmint essential oil (SEO) is known for its potential antioxidant and antimicrobial effects; nevertheless, its food applications are limited due to the volatile nature of its active components. Thus, edible films loaded with essential oil can be an alternative to synthetic preservatives to improve their food applications. In the present study, the effect of SEO addition was investigated on the physicochemical properties of bovine and porcine gelatin films, and antioxidant activity was assessed. GCMS (Gas chromatography mass spectrometry) analysis revealed the presence of carvone (55%) and limonene (25.3%) as major components. The incorporation of SEO into the films decreased the opacity, moisture content, water solubility, and elongation at break of bovine and porcine gelatin films. However, with the addition of EO, the thickness and water vapor permeability of bovine and porcine-based gelatin films increased. Moreover, the addition of SEO increased the tensile strength (TS) of the porcine-based film, whereas bovine samples demonstrated a decrease in tensile strength. XRD (X-ray diffraction) findings revealed a decrease in the percentage crystallinity of both types of gelatin films. SEM (scanning electron microscope) results showed the changes in the morphology of films after the addition of SEO. Antioxidant properties significantly increased with the incorporation of EO (*p* < 0.05) when compared with control films. Therefore, the addition of SEO to gelatin-based edible films could be an effective approach to prepare an active food packaging material to prevent food oxidation.

## 1. Introduction

Active edible materials are soluble thin layers that coat or wrapped around the surface of food to improve and preserve its quality and shelf life by preventing oxidation, microbial contamination, and transmission of gases, moisture, and solutes [1]. Animal-based proteins including gelatin, keratin, whey protein, collagen, and casein present better nutritional, barrier, and mechanical properties than carbohydrate- or lipid-based films [2].

Gelatin is an animal-based biopolymer derived from different sources such as pigs, cows, cattle, and fish. This non-toxic, water-soluble, biodegradable, FDA-approved proteinaceous polymer is derived from collagen, the most abundant protein in mammals [3]. Gelatin has displayed good gel- and film-forming and emulsifying properties with wide applications in the pharmaceutical and food sectors. The quality of gelatin varies according to the viscosity and strength of the gel. Gel strength, often referred to as the “bloom” value, represents the stiffness and strength of the gelatin. It indicates the average molecular weight of gelatin, usually between 30 and 300 bloom (less than 150 for low bloom, between 150–220 for medium bloom, and between 220–300 for high bloom). In addition, the bloom value is directly related to the strength of the gelatin, i.e., a higher bloom value indicates greater gelatin strength [4].

Recent studies have shown a growing interest in using gelatin in the form of edible films for food packaging. Fakhouri et al. reported starch/gelatin based composite material for the preservation of red crimson grapes [5]. More recently, Jridi et al. reported promising results for cheese preservation using fish-based gelatin films [6]. Moreover, the concept of smart edible films is being actively studied to detect freshness and quality of packaged food. Musso et al. reported gelatin/curcumin-based smart edible films, developed to detect pH change in food [7].

Due to its abundance of availability and low cost, gelatin can be an excellent material for developing active edible films. A chief advantage of using gelatin is that it differs from hydrocolloids because of its digestible properties, and contains the complete set of essential amino acids, except for tryptophan [8]. However, the film-forming property is influenced by the source and type of gelatin. Additionally, the amino acid and imino acid composition, along with the degree of prohydroxylation, plays a major role in defining the viscoelastic properties of gelatin. The amino acid sequence and composition of gelatin vary from one source to another. However, all sources always contain good amounts of glycine, proline, and hydroxyproline. Porcine-derived gelatin showed higher levels of glycine, proline, and arginine as compared to bovine gelatin. Moreover, the bloom strength of porcine gelatin was reported to be significantly more than bovine gelatin in the pH range of 3–10. However, the polypeptide pattern among gelatin derived from both sources is very similar [9]. Moreover, the average molecular weight distribution of various components of collagen, i.e., α- chains, β- or γ-components and the α1- and α2-chain ratios, are identified as key elements for the determination of the gelling properties [10].

Therefore, in order to compare their gelling properties, porcine and bovine gelatin were selected for this study. However, gelatin-based films present poor water barrier and mechanical properties when compared to plastic material. The incorporation of plant-based essential oils into gelatin-based edible coating material can improve its barrier properties against water vapor [11]. Therefore, spearmint essential oil was incorporated to evaluate its impact on the physicochemical properties of porcine and bovine gelatin.

Spearmint belongs to the genus Mentha in the family *Labiatae* (*Lamiaceae*) [12]. The spearmint essential oil (SEO) is derived from *Mentha spicata* and it has showed antimicrobial and antioxidant properties which are worth studying in an edible biofilm. The main bioactive compounds present in SEO include carvone and limonene [13]. These biologically active compounds play a major role in antispasmodic, diuretic, antimicrobial, and antioxidant properties [14]. The incorporation of SEO into polymeric edible films can be useful for the development of biologically active films. Moreover, the abundant availability of SEO makes it an excellent candidate to develop sustainable, cost-effective and ecofriendly packaging material.

The current study primarily aimed to evaluate and compare the film-forming properties of two different types of gelatins, i.e., porcine (Po) and bovine (Bo). In addition, we also studied the effects of spearmint essential oil on these films and study their antioxidant properties.

## 2. Materials and Methods

### 2.1. Chemicals

Tween 80, pure porcine and bovine gelatin were purchased from Sisco Research Laboratories Pvt Ltd. (Mumbai, India). The spearmint essential oil (SEO) was supplied by Nature Natural India (Batch No: NNISPEO/213/0921). The glycerol (99.0% pure) used in film preparation was purchased from BDH Laboratory Supplies, (London, UK).

### 2.2. Edible Films (EFs) Preparation

The casting method was employed for the fabrication of porcine- and bovine-based films. A total of eight film samples (four porcine and four bovine samples) were prepared, out of which six, i.e., Po2, Po3, Po4 (porcine), and Bo2, Bo3, Bo4 (bovine) were loaded with various concentrations of SEO. Moreover, two samples were without SEO (Po1 for porcine and Bo1 for bovine), which were considered as the control samples. In order to prepare a 2% (*w*/*v*) solution, 2 g of both Po and Bo were added to 100 mL of distilled water. The solutions were stirred at 60 °C using a magnetic stirrer until completely dissolved. The resultant solutions were divided equally into four parts (20 mL) and labeled as Po1–Po4 for porcine-based films, and Bo1–Bo4 for bovine-based EFs. Subsequently, 0.3% glycerol was added to each sample as a plasticizer as per the volume of the solution. Later, SEO was added in several concentrations (0.05%, 0.075%, and 0.1%) with an equal proportion of Tween 80 (Table 1). Finally, the resultant film-forming solutions were cast into Petri dishes and dried for 48 h at room temperature. After the complete drying of the samples, all of them were peeled. The films were stored in plastic bags and kept at 50% relative humidity in desiccators for 24 h before further experimentation. All the experiments were carried out in triplicate.

### 2.3. Gas Chromatography Mass Spectrometry (GCMS) Analysis

Gas Chromatography Mass Spectrometry (GCMS) analysis of spearmint essential oil (SEO) was performed using a GCMS-QP-2010 Plus Gas chromatograph Mass Spectrometer (Shimadzu, Japan). After injecting the sample, the oven was maintained at a temperature of 50 °C (2 min) and later programmed to 210 °C (ramp rate: 3 °C/min). Further increase in temperature (280 °C) was achieved by increasing the ramp to 6 °C/min. 1 µL of the sample (SEO) was injected (split ratio = 1:115). Helium was utilized at a consistent flow rate (1.0 mL/min) as the carrier gas. The temperatures of the ion source and injector were set at 220 and 260 °C, respectively. The compounds were identified by comparing the retention indices and retention times (RT) of the chromatographic peaks with the reference standards run under identical conditions. Peak enrichment was also performed on co-injection with authentic reference compounds. The comparison of the MS fragmentation pattern with those of pure compounds and mass spectrum database search was performed using the National Institute of Standards and Technology (NIST) MS spectral database (version 2005).

### 2.4. X-ray Diffraction (XRD)

The Bruker D8 Discover instrument was utilized for the characterization of the XRD patterns of the EFs. The instrument was operated at 40 kV with the range of 2θ set between 5 to 50°.

### 2.5. SEM (Scanning Electron Microscopy) Analysis

Surface morphology along with cross-sectional morphology of the edible films was assessed by utilizing JSM6510LA, Analytical SEM, Jeol, Japan. The operation of the instrument was performed at 20 kV. Manual fracture of all the films was performed in liquid nitrogen and mounted on aluminum stubs using adhesive tape and before taking images, the samples were sputter-coated with a thin layer of gold.

### 2.6. FTIR Spectra Analysis

The FTIR Spectrometer (InfraRed Bruker Tensor 37, Ettlingen, Germany) was employed for the characterization of the chemical structures of EFs. The instrument was set up with an attenuated total reflection (horizontal) device over a range of 400 to 4000 cm^−1^ with 4 cm^−1^ resolution and 32 scans.

### 2.7. Mechanical Properties

The assessment of the film’s mechanical properties was carried out in accordance with the ASTM D882 (American Society for Testing and Materials. ASTM., 2010) standard methods by utilizing a load cell (5 kg) with a Universal Tester (TA.XT plus, Stable Micro Systems, Surry, England). Prior to conducting these parameters, we performed film preconditioning in a test cabinet (Nüve TK 120, Ankara, Türkiye) for at least 40 h at a temperature of 25 °C at 50% relative humidity (RH). Afterwards, the strips of film were cut with dimensions of 7 mm width and 60 mm length. The analysis of these strips was performed at 30 mm/min. The assessment of the mechanical properties of the films was carried out in accordance with the elongation at break (%) and Tensile strength (MPa). The equations below were employed for the calculation of tensile strength (TS) and elongation at break (EAB), respectively:

$$\mathrm{TS}=\left(\frac{F}{A}\right)$$

where,

 F = maximum force
 A = cross-sectional area of the film

$$\mathrm{EAB}\;\left(\%\right)=\frac{\mathrm{Lf}-\mathrm{Li}}{\mathrm{Li}}\times 100$$

where,

 Lf = final length at a break
 Li = initial length of the film

### 2.8. Film Thickness

The digital micrometer (Yu-Su 150, Yu-Su Tools, Shanghai, China) was utilized to measure the GE–SEO film thickness with an accuracy of 0.01 mm. The readings were taken from 5 different sites and the mean value was calculated for each film.

### 2.9. Color Analysis

The color analysis was carried out as per the methodology described by Rhim et al. [15]. A colorimeter with a white calibration plate was used (Minolta Colorimeter CR-300, Minolta Camera Co., Osaka, Japan). Film fragments 3 cm in diameter were evaluated according to the Hunter scale (L*, a*, and b*). This experiment was performed in duplicate. Based on data obtained from L*, a*, and b*, the color difference (ΔE) was calculated using the following formula:

$$\Delta E=\sqrt{\Delta L^{2}+\Delta a^{2}+\Delta b^{2}}$$

where,

 ΔL = L standard − L sample; Δa = a standard − a sample;
 Δb = b standard − b sample.

### 2.10. Opacity

The opacity of the GE–SEO edible films was measured according to the spectrophotometric method (ONDA-Vis spectrophotometer, V-10 Plus, ONDA, Padova, Italy) employed by Zhao et al. [16]. After cutting the films, they were placed in cuvettes and opacity was measured by employing a spectrophotometer at a wavelength of 550 nm. The following equation was utilized to measure the GE–SEO film transparency:

$$\mathrm{Opacity}=\left(\frac{A_{550}}{x}\right)$$

where,

 A_550_ = absorbance at 550 nm; x = film thickness (mm).

### 2.11. Moisture Content (MC)

The MC of the films was calculated by employing a modified version of the method used by Erdem et al. [17]. The 3 × 4 cm film sections were cut and weighed and the W_1_ (initial weight) was noted. The drying of the films was performed at 105 °C until we achieved a constant weight and noted W_2_ (final weight). The following equation was employed to calculate the MC:

$$\mathrm{MC}=\frac{W_{1}-W_{2}}{W_{1}}\times 100$$

where,

 W_1_ = initial weight; W_2_ = final weight.

### 2.12. Water Solubility (WS)

The WS of the films was evaluated by utilizing a modified version of the method of Kim and Song [18]. The drying was carried out at a temperature of 105 °C using an oven after cutting the GE–SEO films into manageable strips of 3 × 4 cm^2^. The drying was performed until a constant weight was achieved, and the dry weight was denoted by W_1_. Subsequently, the dried strips were mixed with distilled water (20 mL) and thorough mixing was performed for 24 h by employing a shaking incubator (IKA KS3000 IC, IKA^®^-WerkeGmbH&Co. KG, Staufen, Germany). The excess water was later drained, and drying of the films was performed in an oven at 105 °C. The reading of final weight was then taken and denoted as W_2_. The following equation was utilized to calculate the WS of these films:

$$\mathrm{WS}=\frac{W_{1}-W_{2}}{W_{1}}\times 100$$

where,

 W_1_ = initial weight; W_2_ = final weight.

### 2.13. Water Vapor Permeability (WVP)

The gravimetric method followed by Erdem et al. [17] was utilized for the calculation of WVP. The Gelatin–Spearmint Essential Oil (GE–SEO) films were subjected to conditioning at 50% RH in a desiccator. We employed cups made up of glass with an internal diameter of 5 cm and a depth of 3 cm. Silica gel (RH = 0%) and water (RH = 100%) were utilized for the adjustment of the RH measurement system. Films were sealed firmly over the cup containing silica gel, and the weight measurement of the cups was conducted periodically (every hour) to assess the weight gain over a single day. The calculation of WVP values was carried out by employing the following formula:

$$\mathrm{WVP}=\frac{\Delta m}{\Delta t\times \Delta P\times A}\times d$$

where,

 WVP = water-vapor permeability (g mm/(m^2^) (d)(kPa)); Δm/Δt = weight of moisture gain per unit of time (g/s); A = the area of film (m^2^); ΔP = water vapor pressure difference between the two sides of the film (kPa); d = film thickness (mm).

### 2.14. Antioxidant Assays

#### 2.14.1. Determination of Scavenging Capacity against DPPH Radicals

The assessment of DPPH free radical scavenging activity was carried out in accordance with the method followed by Brand-Williams et al. [19]. First, 50 mg of film sample was vortexed with 1.95 mL of DPPH solution for 30 s and then incubated for 30 min in dark room conditions. The average of three measurements of blank and film sample absorbance was taken at 517 nm and the results were expressed as % absorbance inhibition. Every test was repeated three times. The calculation of percentage inhibition was performed by using the following formula:

Percentage inhibition (%) = (Ac − At)/(Ac) × 100

where,

 Ac represents the absorbance of the blank solution and At indicates the absorbance of the test solution.

#### 2.14.2. Determination of Scavenging Capacity against ABTS Radicals

The method employed by Re et al. [20] was utilized for the evaluation of the ABTS free radical scavenging capacity of films with a few modifications: the change in absorbance was measured kinetically at 734 nm for a period of 6 min. This procedure was performed on a vortexed solution (30 s) of the film (25 mg) and 1.9 mL of 7 mmol/L ABTS radical solution produced with potassium persulfate solution (2.45 mM).

### 2.15. Statistical Analysis

Each result is illustrated as the mean value ± standard deviation (S.D.) of three independent replicates. One-way analysis of variance and Duncan’s test were carried out to analyze the significance of differences between mean values at the 5% level of significance using Minitab 17 software.

## 3. Results and Discussion

### 3.1. Chemical Composition of Spearmint Essential Oil (SEO)

**Figure 1:**
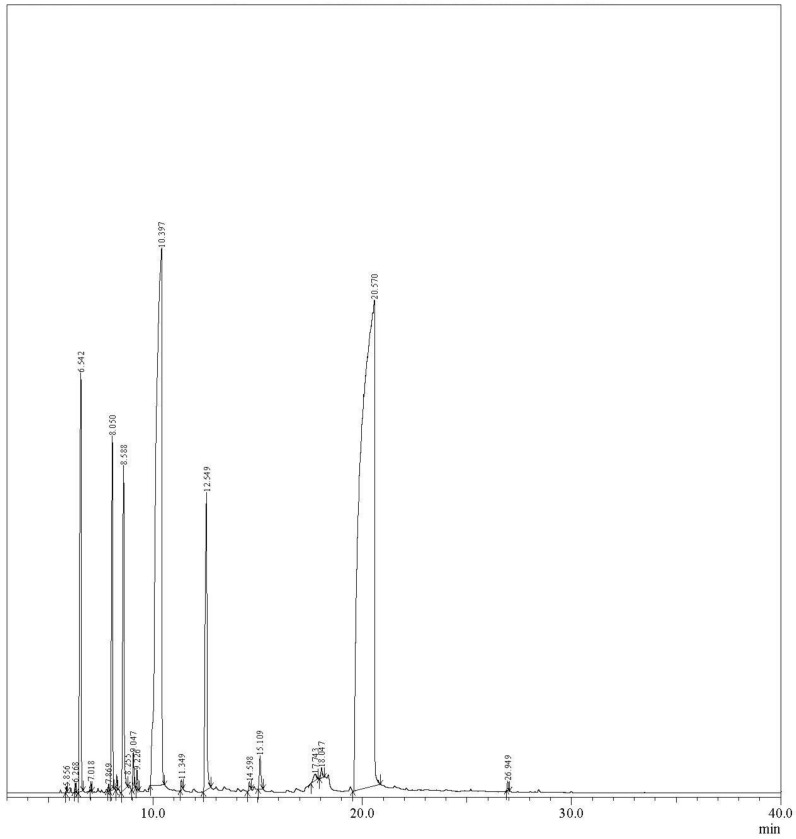
GCMS chromatogram of spearmint essential oil (SEO).

Based on the GCMS (Gas chromatography mass spectrometry) analysis, the major components of spearmint essential oil (SEO) identified in this study are illustrated in Table 2. The components identified in the current study are in alignment with the findings of a previous study [14]. A total of 19 components were identified in essential oil (EO) sample. The major components identified were carvone (55.01%) and limonene (25.38%), with other minor components including α-pinene (5.25%), cyclohexene (4.61%), β-pinene (3.97%), and β-myrcene (3.91%). Figure 1 shows the GC–MS chromatogram of SEO. Another study also reported that similar components including carvone, menthone, limonene, and 1,8-cineole were found as major components in SEO [21]. Similarly, another study revealed that the wild linalool-rich chemotype of spearmint in Greece contained linalool as the main component of the essential oil [22]. These variations in the concentration and composition of different compounds can be attributed to different climatic conditions and geographical locations as well as metabolism, maturity, and the part of the plant from which the EO is extracted [23].

### 3.2. SEM

**Figure 2:**
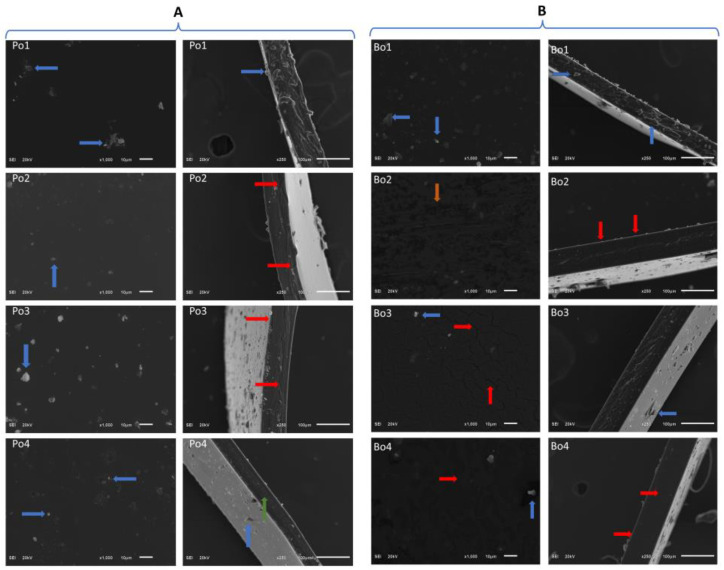
(**A**) Surface and cross-sectional SEM analysis of gelatin (porcine) films loaded with SEO. Po1: Po-control; Po2: Po-SEO (0.05% *v*/*v*); Po3: Po-SEO (0.075% *v*/*v*); Po4: Po-SEO (0.1% *v*/*v*). (**B**) Surface and cross-sectional SEM analysis of gelatin (bovine) films loaded with SEO. Bo1: Bo-control; Bo2: Bo-SEO (0.05% *v*/*v*); Bo3: Bo-SEO (0.075% *v*/*v*); Bo4: Bo-SEO (0.1% *v*/*v*). The arrows represent different properties i.e., Red: cracks, Blue: particles, Green: compactness, Orange: irregular patches.

Surface morphology is significantly influenced by the inherent attribute of the components present in the films and their respective interactions with each other, as well as the conditions in which films are processed [24]. Therefore, SEM analysis was carried out to determine the correlation between the morphological features of films with their respective compositions. Figure 2A and B illustrate the surface and cross-sectional images of porcine (Po1–Po4) and bovine (Bo1–Bo4) films, respectively. The control films show good structural behavior as compared to the EO-loaded films. The EO-loaded films were observed to have developed cracks due to the incorporation of EO. In the case of porcine-based films, few cracks were observed with increase in the SEO concentration. However, in bovine-based films, the cracks were more pronounced due to the addition of SEO. Similar results were reported by Sancakli et al., who studied the effect of plasticizers on the physicochemical properties of gelatin films [25].

### 3.3. XRD Analysis

**Figure 3:**
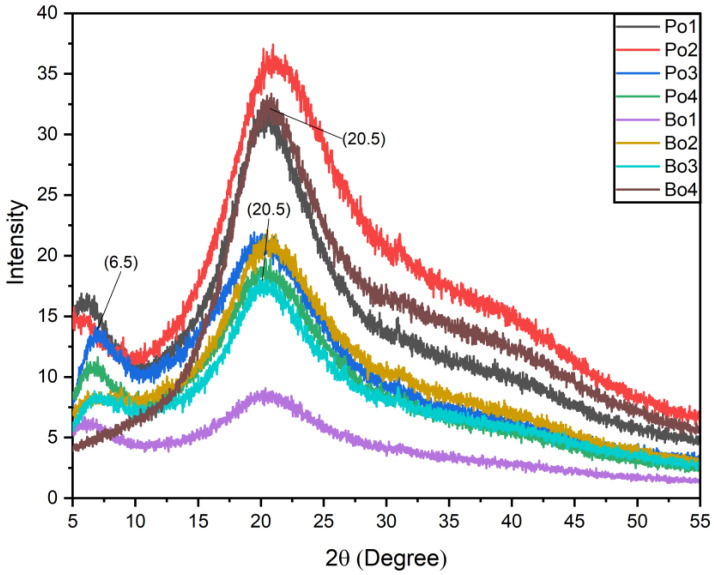
XRD comparison of two types of gelatin (Porcine and Bovine) films loaded with SEO. Porcine-based films: Po1: Po-control; Ppo2: Po-SEO (0.05% *v*/*v*); Po3: Po-SEO (0.075% *v*/*v*); Po4: Po-SEO (0.1% *v*/*v*). Bovine-based edible films: Bo1: Bo-control; Bo2: Bo-SEO (0.05% *v*/*v*); Bo3: Bo-SEO (0.075% *v*/*v*); Bo4: Bo-SEO (0.1% *v*/*v*).

Figure 3 illustrates the XRD diffractograms of porcine and bovine-based gelatin films incorporated with various concentrations of SEO. The diffractograms of all films exhibited similar diffraction patterns with variations in the intensities. X-ray diffractograms present diffraction peaks between 6–8° and 20° in all the samples except Bo4. The characteristic peak between 6–8° could be due to the formation of the triple-helix structure of gelatin [26]. A single broad peak was observed at 20° in Bo4 (film-loaded maximum concentration of SEO). This could be due to the alteration of the triple-helix structure upon the addition of a high concentration of SEO, which has been reported in other studies using various additives [27].

Furthermore, the addition of SEO impacted the % crystallinity of the EFs. A decrease in overall crystallinity was observed in both types of gelatin. The crystallinity of Po-based EFs decreased from 39.2% (control) to 30% (film loaded with maximum concentration of SEO), whereas the crystallinity of Bo-based EFs decreased from 35% (control) to 26.5% (film loaded with maximum concentration of SEO). The % crystallinity is different in both types of gelatin. The degree of crystallinity depends upon the content of triple helical structures in gelatin. The content of triple helical structures depends on the source animal of the gelatin, and it plays an essential role in defining the mechanical properties of the films [28]. Moreover, Bergo and Sobral have reported that the addition of glycerol increases the amorphous nature of gelatin-based films regardless of the type of gelatin [29]. However, in the current study, a further increase in the amorphous nature of the EFs was observed, which could be due to SEO addition.

### 3.4. FTIR Spectroscopy

**Figure 4:**
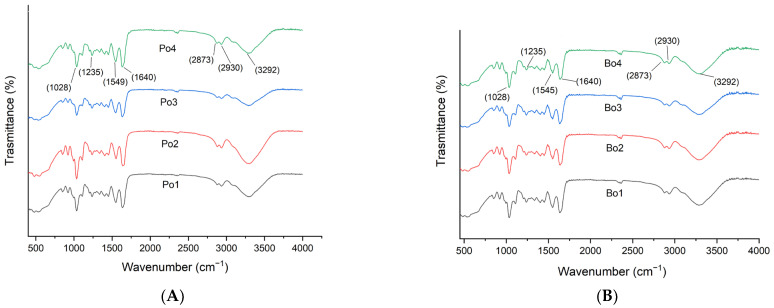
FTIR analysis of SEO-loaded gelatin films. (A) Porcine-based films: Po1: Po-control; Po2: Po-SEO (0.05% *v*/*v*); Po3: Po-SEO (0.075% *v*/*v*); Po4: Po-SEO (0.1% *v*/*v*). (B) Bovine-based edible films: Bo1: Bo-control; Bo2: Bo-SEO (0.05% *v*/*v*); Bo3: Bo-SEO (0.075% *v*/*v*); Bo4: Bo-SEO (0.1% *v*/*v*).

Figure 4A,B illustrate the FTIR spectra of porcine (Po) and bovine (Bo) films, respectively. Both overlays show similar peak patterns. The peak observed at the wavelength 1033 cm^−^^1^ in each sample could be due to glycerol [30]. Furthermore, the observation of spectral peaks revealed various regions and functional groups in our samples. The Amide I was observed at wavenumber 1640 cm^−^^1^ (C=O stretching vibrations), while Amide II and Amide III were observed at 1549 cm^−^^1^ (N–H bending vibrations) and 1239 cm^−^^1^ (C–N stretching vibrations), respectively [31]. The peak at 3070 cm^−^^1^ indicates Amide B, whereas the peak at 3292 cm^−^^1^ represents Amide A produced due to stress vibrations in the N-H group [25]. Similar results were also reported in a previous study conducted on bovine-based active films [32]. However, the addition of EOs and other additives usually causes shifts and changes in the amplitudes of amide bands [33,34].

Additionally, it was observed that the SEO-loaded films did not display any additional peaks. However, changes in peak intensities were observed, which can be due to interaction between some functional groups of SEO and gelatin. Similar results were reported in previous studies where no clear chemical change was observed due to the addition of essential oils (fennel and peppermint essential oil) in chitosan films [35].

### 3.5. Mechanical Properties

In terms of structural integrity, mechanical features play an important role in determining the durability and cohesion of edible films. The mechanical properties, i.e., tensile strength (TS) and elongation at break (EAB), of the edible gelatin films with different concentrations of spearmint essential oil (SEO) are illustrated in Table 3. The results indicate that porcine-based films incorporated with SEO (0.05, 0.075, and 0.1% *v*/*v*) displayed an increase in the TS and a decrease in the EAB. The TS increased from 9.25 to 24.46 MPa, whereas the EAB decreased from 197.5 in control to 43.54 in 0.1% SEO-loaded porcine film. The increase in TS was probably due to the formation of a more compact film matrix due to the increase in SEO concentration. This can be due to the formation of a strong protein network in which SEO molecules were homogenously entangled within porcine gelatin molecules [36]. The SEM analysis (Figure 2A) of porcine gelatin supports this observation, as a compact film structure was observed as the concentration of SEO was increased.

On the other hand, we observed a decrease in the TS as well as the EAB with an increase in SEO concentration in bovine-based EFs (Table 3). A similar trend of decreasing TS was observed in another study where bovine-based edible films were incorporated with essential oil [32]. This behavior could be due to the hindrance of the gelatin chain interaction, leading to decreased TS of bovine-based films. The difference in the TS of films fabricated from different sources of gelatin is probably due to a variation in their triple helical content. The films containing a higher content of triple helical structures show more crystalline behavior, which in turn improves the TS of films [37]. In this study, we observed that the porcine-based films had much higher crystallinity and TS as compared to the bovine films.

### 3.6. Thickness

The results of thickness are illustrated in Table 3. The films displayed a gradual increase in thickness with the increase in essential oil concentration. The thickness of the porcine films was observed to increase slightly from 0.023 mm in the control to 0.048 mm at the maximum concentration of spearmint essential oils (0.1%). However, the bovine gelatin displayed considerable variation in thickness with the increase in SEO concentration. We observed that the thickness increased from 0.052 mm in the pure bovine films to 0.1 mm in EFs incorporated with 0.1% SEO. Similar results were observed for fish gelatin films incorporated with various EOs. The increase in the thickness of the films with SEO can be due to the increase in solid content, or the presence of SEO might hinder peptide chain formation in gelatin [33].

### 3.7. Color Analysis and Opacity

The visual attributes, i.e., color and transparency of edible films are essential due to their use in the food industry as packing material [38]. The results illustrated in Table 4 indicate that the L* value (lightness) decreased with the addition of SEO. However, the change in the L* value was not significant, and similar results were observed in both Po- and Bo-based films. The yellowness of the EFs increased with the increase in the concentration of SEO (b* values). The yellowness of the porcine-based EFs increased from 1.60 (control) to 5.43 (emulsified films with maximum concentration of SEO). The bovine-based EFs also displayed an increase in yellowness from 1.34 (control) to 2.31 (emulsified films with maximum concentration of SEO). However, the change in color in the bovine films was not as significant as compared to the porcine films.

In addition, a decrease in opacity was observed due to the incorporation of SEO. The opacity of porcine-based films was observed to decrease from 2.03% (control) to 0.78% (maximum concentration of SEO). The bovine-based films also showed similar results whereby opacity decreased from 0.61% (control) 0.13% (maximum concentration of SEO). According to the absorbance measurements, the decrease in film opacity means an increase in transparency. Similar results were observed in another study, where gelatin films loaded with beeswax and carnauba wax displayed an increase in transparency [39].

### 3.8. Moisture Content (MC)

The moisture content of gelatin edible films can vary depending on the specific production method and storage conditions. The moisture content of our samples is illustrated in Table 3. It was observed that the moisture content of both types of gelatin films decreased with the addition and increase in SEO concentration. The MC of porcine-based EFs decreased from 37.89% in control films to 15.20% in 0.1% SEO loaded films. Similar results were observed in bovine-based edible films, where a drop in MC was observed from 35.06% (control) to 16.13% (maximum concentration of SEO). The reason for this drastic decrease in MC can be due to the hydrophobic and water-repellant nature of essential oils, as a result of which the increase in SEO concentration results in lower MC. Similar results were observed in a previous study, where the increase in the concentration of oil resulted in a decrease in the moisture content of the EFs [40]. A recent report published by Shen et al. reported similar results, where the hydrophobic nature of clove essential oil resulted in a decrease in the moisture content of the pullulan–gelatin composite films [41]. Another study conducted by Liu et al. reported similar results with Konjac glucomannan films loaded with thyme essential oil [42].

### 3.9. Water Solubility (WS)

Water solubility is an important property of edible films as it can affect the functionality and shelf life of the film. High water solubility can be useful for certain applications where the film needs to dissolve quickly to release the product, such as coatings or packaging for fresh products. On the other hand, insoluble films are useful for applications where the water-resistant film needs to maintain its integrity and shape, such as in candy or snack packaging. The solubility of a film can be influenced by factors such as the type and concentration of polymers, as well as the presence of other additives and the processing conditions. The present results are illustrated in Table 3. The slight decrease in the solubility of the films is due to an increase in the concentration of SEO. The WS (%) of porcine-based EFs decreased from 69.45% (control) to 62.19% (emulsified EFs with maximum concentration of SEO), whereas the WS (%) of Bovine based EFs decreased from 56.42% (control) to 47.66% (emulsified EFs with maximum concentration of SEO). The decrease in MC could be due to the hydrophobic effect of the SEO. A recent report published by Anis et al. suggested that the incorporation of essential oils significantly reduced the WS (%) of films due to their hydrophobic nature [43]. Similar results were reported in a recent study where a decrease in WS was observed in gelatin–alginate composite films upon the addition of Anise essential oil [44].

### 3.10. Water Vapor Permeability

Water vapor permeability is an important trait for edible films, as it allows for the proper management of moisture in the packaged food product. A film with high water vapor permeability allows the transmission of excess moisture, which can impact the quality and extend the shelf life of the food. On the other hand, a film with low water vapor permeability can restrict water vapor transmission. Therefore, a good edible film should have moderate water vapor permeability. In the present study, an increase in the WVP of the EFs was observed with the increase in the concentration of SEO. The results are illustrated in Table 3. The literature reports similar results in another study where the use of bergamot oil (BO) resulted in an increase in WVP in gelatin films [11].

The possible reason for a considerable increase in WVP of bovine-based films could be physical damage caused to the films due to the addition of SEO. The SEM analysis revealed a substantial increase in cracks as the concentration of SEO was increased. Similar results were reported in a previous report, where cinnamon essential oil was used as a bioactive compound in chitosan- and cellulose-based films [45]. Another recently reported study by Saranti et al. where an increase in WVP was observed due to physical damage caused to the films due to the incorporation of black pepper essential oil in gelatin-based films [46].

### 3.11. Radical Scavenging Activity

**Figure 5:**
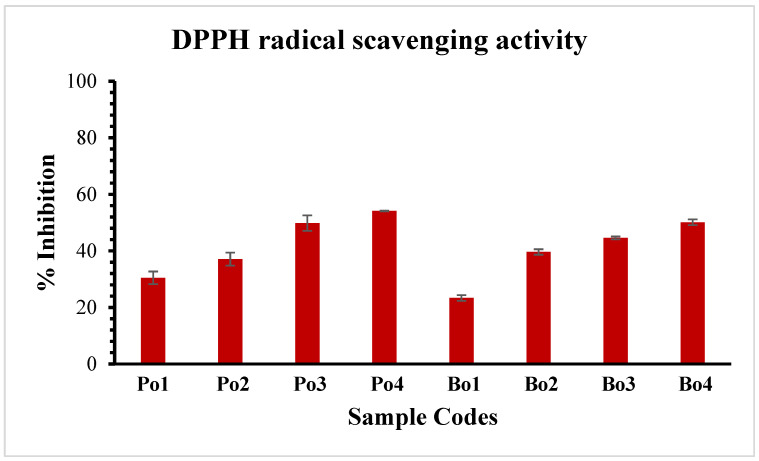
Illustration of DPPH radical scavenging activity of gelatin (Po and Bo) edible films loaded with SEO. Porcine edible films: Po1: Po-control; Po2: Po-SEO (0.05% *v*/*v*); Po3: Po-SEO (0.075% *v*/*v*); Po4: Po-SEO (0.1% *v*/*v*). Bovine edible films: Bo1: Bo-control; Bo2: Bo-SEO (0.05% *v*/*v*); Bo3: Bo-SEO (0.075% *v*/*v*); Bo4: Bo-SEO (0.1% *v*/*v*). Error bars display standard deviation.

**Figure 6:**
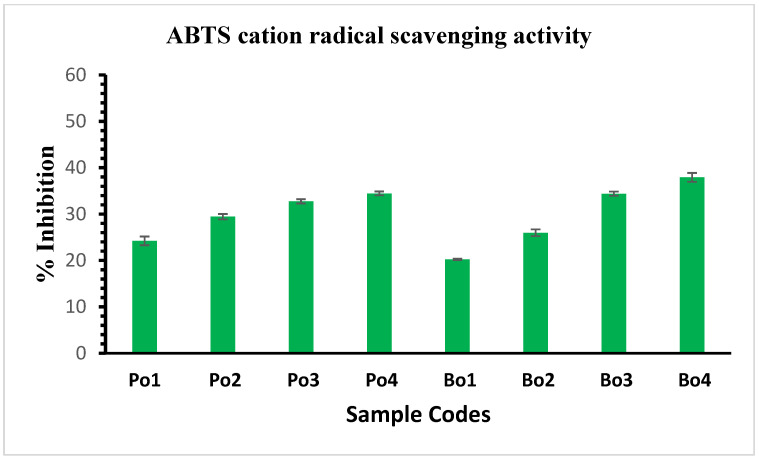
Illustration of ABTS cation radical scavenging activity of gelatin (Po and Bo) edible films loaded with SEO. Porcine edible films: Po1: Po-control; Po2: Po-SEO (0.05% *v*/*v*); Po3: Po-SEO (0.075% *v*/*v*); Po4: Po-SEO (0.1% *v*/*v*). Bovine edible films: Bo1: Bo-control; Bo2: Bo-SEO (0.05% *v*/*v*); Bo3: Bo-SEO (0.075% *v*/*v*); Bo4: Bo-SEO (0.1% *v*/*v*). Error bars display standard deviation.

In this study, we employed DPPH and ABTS radical scavenging assays to study the antioxidant potential of the active films. Figure 5 and Figure 6 illustrate the DPPH and ABTS free radical scavenging activity, respectively. A dose-dependent increase in antioxidant activity was observed, with the maximum activity observed at the highest concentration of SEO. The GCMS analysis revealed the presence of monoterpenoids in SEO, i.e., carvone, which plays a vital role in the antioxidant activity of the films [47]. Additionally, the control films also revealed slight antioxidant activity, which could be due to the presence of certain peptide fractions and amino acids such as glycine and proline [48,49]. Moreover, the porcine-based films displayed slightly better free radical scavenging results in comparison to the bovine-based edible films. This can be due to variations in the amino acid residues of both sources of gelatin.

## 4. Conclusions

In the present work, lower concentrations of SEO were used to prepare active food packaging material using gelatin from two different sources: bovine and porcine. The incorporation of SEO in the filmogenic solutions caused changes in the physical and chemical properties of the resulting gelatin films. The most significant changes were observed in EAB, TS, WS, WVP, opacity, and thickness values. On the other hand, the addition of SEO adversely influenced the morphology of the films by creating cracks. Furthermore, XRD findings revealed a decrease in the percentage crystallinity of both types of gelatin film. The addition of SEO significantly (*p* < 0.05) increased the antioxidant properties of films when compared with control films. Thus, based on the present findings, it was found that the addition of SEO could be an effective approach for the preparation of gelatin-based active films, which can be later used to preserve several food products. Additionally, the results give a clear indication that the porcine-based gelatin films displayed better tensile strength and other barrier properties due to incorporation of SEO as compared to bovine-based gelatin.

## Figures and Tables

**Table 1:** Chemical composition of SEO-loaded gelatin (Po and Bo) films.

Sample Codes	Chemical Composition
Po1	GE (Po) (2%) + Gly (0.3%)
Po2	GE (Po) (2%) + Gly (0.3%) + SEO (0.05%) + Tween 80 (0.05%)
Po3	GE (Po) (2%) + Gly (0.3%) + SEO (0.075%) + Tween 80 (0.075%)
Po4	GE (Po) (2%) + Gly (0.3%) + SEO (0.1%) + Tween 80 (0.1%)
Bo1	GE (Bo) (2%) + Gly (0.3%)
Bo2	GE (Bo) (2%) + Gly (0.3%) + SEO (0.05%) + Tween 80 (0.05%)
Bo3	GE (Bo) (2%) + Gly (0.3%) + SEO (0.075%) + Tween 80 (0.075%)
Bo4	GE (Bo) (2%) + Gly (0.3%) + SEO (0.1%) + Tween 80 (0.1%)

GE: Gelatin, Po: Porcine, Bo: Bovine, Gly: Glycerol, SEO: Spearmint essential oil.

**Table 2:** The main chemical components of Spearmint essential oil (SEO).

Component	Area (%)	RT *
Carvone	55.01	20.570
Limonene	25.38	10.397
α-Pinene	5.25	6.542
Cyclohexene	4.61	12.549
β-pinene	3.97	8.050
β-Myrcene	3.91	8.588

* Retention time (min).

**Table 3:** Tensile strength (TS) and elongation at break (EAB), water solubility (WS), water vapor permeability (WVP), moisture content (MC) and thickness of porcine (Po1–Po4) and bovine (Bo1–Bo4) edible films loaded with SEO.

Sample Codes	TS (MPa)	EAB (%)	WS (%)	WVP	MC (%)	Thickness (mm)
Po1	9.25 ± 0.15 ^d^	197.85 ± 5.18 ^b^	69.45 ± 0.40 ^a^	0.167 ± 0.01 ^a^	37.89 ± 1.08 ^a^	0.023 ± 0.005 ^a^
Po2	12.15 ± 0.68 ^c^	186.32 ± 1.86 ^c^	65.96 ± 0.77 ^b^	0.220 ± 0.01 ^b^	35.04 ± 1.00 ^b^	0.027 ± 0.005 ^a^
Po3	14.03 ± 0.38 ^b^	170.42 ± 4.24 ^d^	64.54 ± 0.25 ^c^	0.245 ± 0.01 ^c^	28.42 ± 1.94 ^d^	0.032 ± 0.016 ^ab^
Po4	24.46 ± 1.85 ^a^	43.54 ± 2.11 ^h^	62.19 ± 0.38 ^d^	0.277 ± 0.00 ^d^	15.20 ± 2.35 ^e^	0.048 ± 0.010 ^b^
Bo1	2.63 ± 0.03 ^e^	226.79 ± 5.41 ^a^	56.42 ± 0.72 ^e^	0.509 ± 0.00 ^e^	35.06 ± 0.82 ^b^	0.052 ± 0.008 ^bc^
Bo2	1.89 ± 0.03 ^f^	131.80 ± 4.06 ^e^	51.86 ± 0.34 ^f^	0.591 ± 0.00 ^f^	32.35 ± 1.18 ^c^	0.066 ± 0.009 ^cd^
Bo3	0.85 ± 0.01 ^g^	106.70 ± 1.79 ^f^	47.71 ± 0.35 ^g^	0.723 ± 0.01 ^g^	27.67 ± 1.11 ^d^	0.082 ± 0.008 ^d^
Bo4	0.84 ± 0.01 ^g^	90.91 ± 2.49 ^g^	47.66 ± 0.98 ^g^	0.771 ± 0.01 ^h^	16.13 ± 2.38 ^e^	0.1 ± 0.008 ^e^

Average values placed in columns marked with different letters (^a–h^) are statistically different.

**Table 4:** Color parameters including L, a*, b*, color difference (ΔE), and opacity of porcine (Po1–Po4) and bovine (Bo1–Bo4) films with spearmint essential oil (SEO).

Sample Code	L	a*	b*	ΔE	Opacity
Po1	95.67 ± 0.06 ^b^	−0.25 ± 0.03 ^cd^	1.60 ± 0.14 ^ef^	0.80 ± 0.15 ^e^	2.036 ± 0.091 ^a^
Po2	95.00 ± 0.27 ^c^	−0.48 ± 0.01 ^a^	3.48 ± 0.19 ^c^	2.81 ± 0.17 ^c^	1.145 ± 0.077 ^b^
Po3	93.46 ± 0.24 ^d^	−0.38 ± 0.02 ^b^	4.30 ± 0.11 ^b^	4.27 ± 0.22 ^b^	0.947 ± 0.045 ^c^
Po4	93.50 ± 0.35 ^d^	−0.36 ± 0.05 ^b^	5.43 ± 0.31 ^a^	5.20 ± 0.23 ^a^	0.779 ± 0.06 ^d^
Bo1	96.02 ± 0.07 ^a^	−0.28 ± 0.02 ^c^	1.34 ± 0.04 ^g^	0.48 ± 0.02 ^g^	0.610 ± 0.014 ^e^
Bo2	95.96 ± 0.09 ^a^	−0.24 ± 0.02 ^d^	1.44 ± 0.02 ^f^	0.57 ± 0.02 _f_	0.265 ± 0.011 ^f^
Bo3	95.99 ± 0.04 ^a^	−0.19 ± 0.01 ^e^	1.81 ± 0.07 ^e^	0.91 ± 0.07 ^e^	0.189 ± 0.009 ^g^
Bo4	95.15 ± 0.06 ^c^	−0.19 ± 0.01 ^e^	2.31 ± 0.13 ^d^	1.66 ± 0.13 ^d^	0.130 ± 0.014 ^h^

Average values placed in columns marked with different letters (^a–h^) are statistically different.

## Data Availability

Not applicable.
